# Supplementary material for: Label-Free Quantitative Proteomic Analysis of Nitrogen Starvation in Arabidopsis Root Reveals New Aspects of H2S Signaling by Protein Persulfidation
Source: Antioxidants (Basel). 2021 Mar 24;10(4):508. doi: 10.3390/antiox10040508 (PMC8064375; doi:10.3390/antiox10040508)
Supplement: Supplementary file 1 [file antioxidants-10-00508-s001.zip › Table S1.docx]

**Table S1. Subcellular distribution of the persulfidated proteins in root tissue**

Location Percentage

cytoplasm 19.6

chloroplast 13.8

ribosome 10.1

cell wall 9.2

Golgi apparatus and ER 9.2

other cellular components 7.5

other intracellular components 6.7

cytoskeleton 5.7

plasma membrane 4.5

extracellular 4.0

nucleus 3.6

other membranes 3.1

mitochondria 2.8

unknown cellular components 0.3
