# Supplementary material for: Label-Free Quantitative Proteomic Analysis of Nitrogen Starvation in Arabidopsis Root Reveals New Aspects of H2S Signaling by Protein Persulfidation
Source: Antioxidants (Basel). 2021 Mar 24;10(4):508. doi: 10.3390/antiox10040508 (PMC8064375; doi:10.3390/antiox10040508)
Supplement: Supplementary file 1 [file antioxidants-10-00508-s001.zip › Figure S1.pdf]

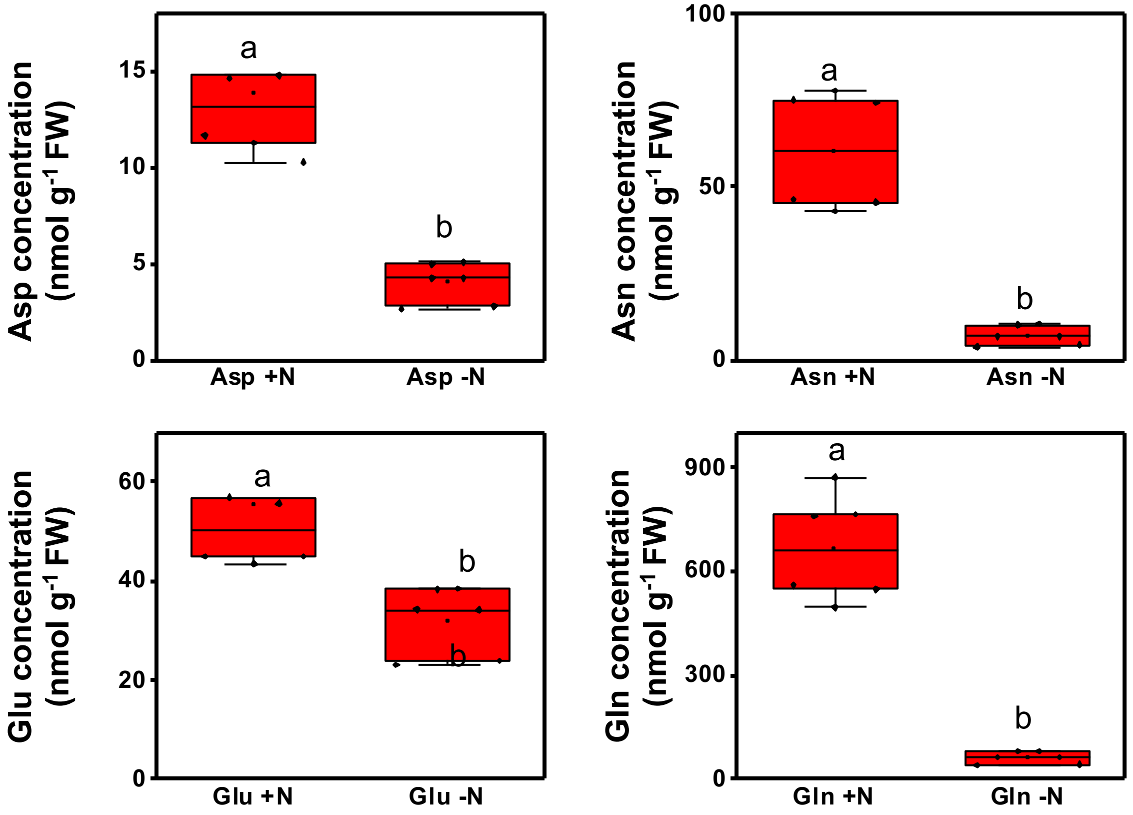

Figure S1. Amino acid determination of Glu, Gln, Asp and Asn in N-sufficient and N-deprived root samples (n = 4). Different letters above the bars indicate significant differences with $P < 0.01$ (One-way ANOVA, post-hoc Tukey adjustment)
